# Supplementary material for: Economic Burden of Respiratory Syncytial Virus Infection in Colombia: A Nationwide Cost-of-Illness Study
Source: J Health Econ Outcomes Res. 2025 Dec 19;12(2):262–9. doi: 10.36469/001c.151678 (PMC12718548; doi:10.36469/001c.151678)
Supplement: Online Supplementary Material [file jheor_2025_12_2_151678_323005.pdf]

Journal of Health Economics and Outcomes Research

ONLINE SUPPLEMENTARY MATERIAL*

Economic Burden of Respiratory Syncytial Virus Infection in Colombia: A Nationwide Cost-of-Illness Study. *JHEOR*. 2025;12(2):262-269. doi:10.36469/jheor.2025.151678

S1. ICD-10 Codes Used to Identify RSV-Related ARI 2
S2. Epidemiological Formulas 2
S2.1. RSV Incidence Rate 2
S2.2. RSV Mortality Rate 2
S3. Extrapolation Procedure for the Subsidized Regime 3
S4. Inflation Adjustment and Currency Conversion 3
S5. Direct Healthcare Cost Components Included 4
S6. Uncertainty Estimation Procedure 4
S7. Analytical Workflow 5
S8. Scenario Considerations and Sensitivity Analyses 5
Supplementary Tables and Figure 6

* This supplementary material has been provided by the authors to give readers additional information about their work.

S1. ICD-10 Codes Used to Identify RSV-Related ARI

The following ICD-10 codes were used to identify respiratory encounters associated with respiratory syncytial virus (RSV) in the UPC database. These codes were selected because they correspond to confirmed RSV infection, RSV-associated lower respiratory tract disease, or acute lower respiratory infections that are clinically consistent with RSV:

- J12.1 – Viral pneumonia due to respiratory syncytial virus
- J20.5 – Acute bronchitis due to respiratory syncytial virus
- J21.0 – Acute bronchiolitis due to respiratory syncytial virus
- J22 – Unspecified acute lower respiratory infection
- B97.4 – Respiratory syncytial virus as the cause of diseases classified elsewhere

Only encounters coded with one or more of these ICD-10 diagnoses were included for the identification of RSV-related ARI in UPC claims.

S2. Epidemiological Formulas

S2.1. RSV Incidence Rate

$$\text{Incidence}_a = \left(\frac{\text{Estimated RSV Cases}_a}{\text{Population at Risk}_a}\right) \times 100{,}000$$

Where a is the age group.

S2.2. RSV Mortality Rate

$$\text{Mortality}_a = \left(\frac{\text{RSV-related deaths}_a}{\text{Population at Risk}_a}\right) \times 100{,}000$$

Death counts were derived from Global Burden of Disease (GBD) estimates and mapped to Colombian age-specific population denominators using SISPRO affiliation data.

S3. Extrapolation Procedure for the Subsidized Regime

National RSV case estimates required extrapolation from the contributory regime (where UPC claims data are available) to the subsidized regime (for which only population denominators are available). The extrapolation followed these steps:

1. Age-specific ARI consultation rate in the contributory regime:

$$Rate_{ARI,a}^{cont} = \frac{ARI_a^{cont}}{Pop_a^{cont}}$$

2. Extrapolation of ARI to the subsidized regime:

$$ARI_a^{subs} = Rate_{ARI,a}^{cont} \times Pop_a^{subs}$$

3. Estimation of RSV cases in the subsidized regime:

$$RSV_a^{subs} = ARI_a^{subs} \times p_{RSV,a}$$

Where $p_{RSV,a}$is the age-specific RSV positivity proportion from sentinel surveillance.

Assumption

Healthcare-seeking patterns and RSV positivity proportions were assumed to be similar across insurance regimes. Potential deviations from this assumption are acknowledged as a limitation in the main manuscript.

S4. Inflation Adjustment and Currency Conversion

All costs recorded in 2019 Colombian pesos (COP) were adjusted to December 2023 COP using the Colombian Consumer Price Index (Health basket), applying:

$$Cost_{2023} = Cost_{2019} \times \frac{CPI_{2023}}{CPI_{2019}}$$

Conversion to U.S. dollars (USD) was performed using the Colombian Representative Market Rate (TRM) for December 31, 2023:

$$Cost_{USD} = \frac{Cost_{2023}}{3{,}822}$$

S5. Direct Healthcare Cost Components Included

The following categories of health services were included, all of which are reimbursed under the National Benefits Plan (Plan de Beneficios en Salud – PBS):

- Emergency department visits
- General outpatient consultations
- Hospitalizations (general ward)
- Intensive care unit (ICU) admissions
- Medications (ATC-classified)
- Laboratory services
- Diagnostic imaging
- Respiratory and physical therapy
- Patient transfers
- Observation unit stays
- All other healthcare services

Indirect costs (e.g., productivity losses, transportation, caregiver time) were not included.

S6. Uncertainty Estimation Procedure

Confidence intervals for cost estimates were calculated using linear propagation of uncertainty, an approach appropriate when the transformation applied to the estimator is deterministic. For each age group and care setting, mean per-patient costs and their 95% confidence intervals were obtained directly from individual-level UPC claims data.

National total costs were computed by multiplying these per-patient means by the estimated number of RSV cases, which is treated as a deterministic scalar. Consequently, the same scalar was applied to the point estimate and to the lower and upper bounds of the per-patient confidence interval:

$$CI_{\text{total}} = CI_{\text{per-patient}} \times \text{Scalar}.$$

This transformation preserves the original uncertainty structure because no additional variance is introduced by the extrapolation factor. No bootstrapping or resampling procedures were used.

S7. Analytical Workflow

The overall analytical workflow followed these steps:

1. Identification of ARI encounters in UPC using ICD-10 codes.
2. Estimation of age-specific RSV positivity proportions using sentinel-confirmed laboratory data.
3. Calculation of contributory-regime RSV-ARI cases.
4. Estimation of contributory ARI consultation rates and extrapolation to the subsidized regime.
5. Application of RSV positivity proportions to extrapolated ARI counts to obtain national RSV case estimates.
6. Extraction of all UPC claims within 30 days of RSV-coded encounters.
7. Aggregation of reimbursed services into standardized cost categories.
8. Inflation adjustment of costs and conversion to USD.
9. Calculation of confidence intervals using linear propagation of uncertainty.
10. Stratification of results by age group and healthcare setting (outpatient care, hospitalization, ICU).

S8. Scenario Considerations and Sensitivity Analyses

A full probabilistic sensitivity analysis was not conducted due to the descriptive cost-of-illness design. However, future extensions should explore:

- Alternative RSV positivity proportions
- Variation in ARI consultation rates
- Age-specific ICU utilization rates
- Potential post-pandemic shifts in respiratory care-seeking behavior

These considerations have been incorporated into the Limitations section of the main manuscript.

Supplementary Tables and Figure

The following tables and figure are included in their original form:

- Table S1. RSV and ARI positivity proportions by age and sex
- Table S2. Estimated RSV cases by insurance regime and age
- Table S3. RSV-related deaths by age
- Figure S1. Proportional distribution of direct healthcare costs among infants under one year
- Figure S2.

Table S1. Proportion of RSV, ARI-Positive Cases

Age Group	Proportion of RSV-Positive Cases			Proportion of ARI-Positive Cases		
	Male	Female	Total	Male	Female	Total
Under 1 year	0.095	0.085	0.090	0.710	0.659	0.685
1 to 4 years	0.069	0.070	0.069	0.319	0.311	0.315
5 to 14 years	0.057	0.068	0.062	0.097	0.096	0.097
15 to 49 years	0.014	0.014	0.014	0.070	0.108	0.090
50 to 74 years	0.019	0.015	0.017	0.063	0.105	0.086
75 years or older	0.013	0.021	0.018	0.100	0.115	0.109
Total	0.048	0.040	0.044	0.092	0.120	0.107

Table S2. Estimated Number of Patients with Respiratory Syncytial Virus Infection Who Sought Healthcare Services in Colombia (2019), Stratified by Age Group and Health Insurance Regime

Age Group	Total Colombia				
	Contributory		Subsidized		Total
	n	%	n	%	
Under 1 year	13,204	42.33%	17,989	57.67%	31,192
1 to 4 years	23,020	41.55%	32,382	58.45%	55,401
5 to 14 years	17,424	41.06%	25,009	58.94%	42,433
15 to 49 years	52,843	51.69%	49,393	48.31%	102,236
50 to 74 years	16,076	52.12%	14,768	47.88%	30,845
75 years or older	1,226	46.51%	1,410	53.49%	2,636
Total	123,793	46.76%	140,951	53.24%	264,744

Table S3. RSV-Related Deaths in Colombia—2019.

Age Group	Male	Female	Total
Under 1 year	119	120	239
1 to 4 years	44	43	87
5 to 14 years	17	14	31
15 to 49 years	43	30	73
50 to 74 years	144	114	258
75 years or older	352	432	784
Total	719	753	1472

Figure S1. Proportional Distribution (%) of Total Direct Healthcare Costs in Children Under 1 Year with RSV infection in Colombia (2019), by Healthcare Service Category

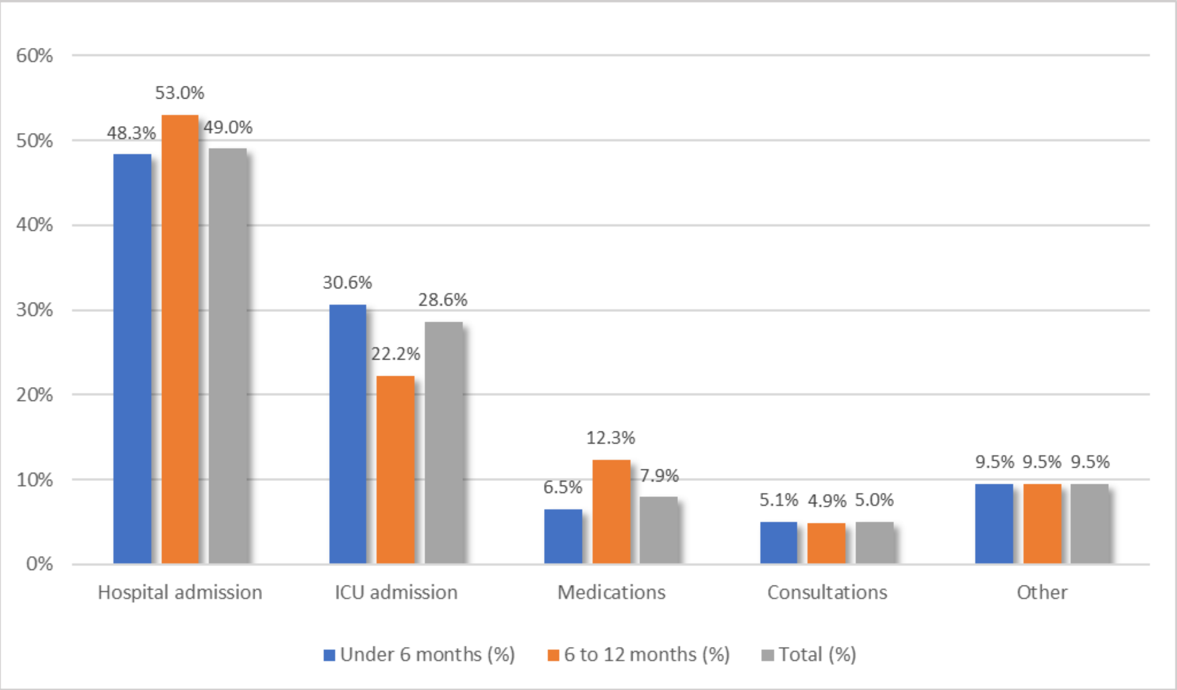

Figure S2. Mortality Related to RSV Infection

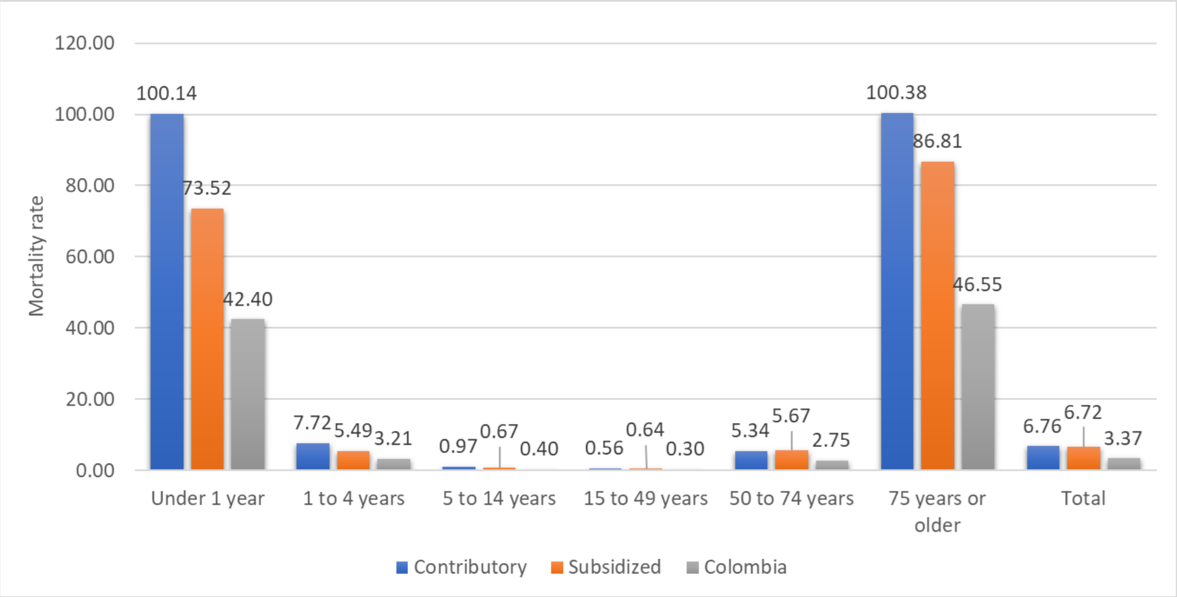

Mortality rates (per 100,000 insured individuals) associated with RSV infection among patients who sought healthcare services in Colombia (2019), stratified by age group.
